# How Older Adults with Chronic Conditions Perceive Artificial Intelligence (AI)-Based Virtual Humans: A Q Methodology Approach

**DOI:** 10.3390/healthcare13131525

**Published:** 2025-06-26

**Authors:** Youn-Gill Jeong, Seo Jung Shin, Song Yi Lee

**Affiliations:** 1 School of Interdisciplinary Studies, Dongguk University-Seoul, 30, Pildong-ro 1 gil, Jung-gu, Seoul 04620, Republic of Korea; 2 Department of Counselling and Coaching, Dongguk University-Seoul, 30, Pildong-ro 1 gil, Jung-gu, Seoul 04620, Republic of Korea

**Keywords:** older adults, artificial intelligence, virtual human, caregiving technology, Q methodology, technology acceptance, continuity theory

## Abstract

**Background/Objectives:** This study examines the subjective perceptions of older adults with mild chronic conditions regarding an artificial intelligence (AI)-based caregiving device, referred to as an “AI Human”, by identifying and categorising their viewpoints through Q methodology. **Methods:** We conducted the study in February 2025 at two adult welfare centres in Buyeo, South Korea. Thirteen older adults used the AI Human device with support for at least 15 days. We initially generated 152 opinion statements through a literature review, focus group interviews, and AI-assisted methods and refined them to a Q sample of 34 statements. Participants completed Q sorts, and we used Ken-Q Analysis software (version 2.0.1) to analyse the data, applying principal component analysis and Varimax rotation. **Results:** Four distinct perception types emerged: (1) emotionally engaged users prioritise reminiscence and emotional interaction; (2) present-oriented conversationalists prefer real-time, everyday dialogue; (3) usage-burdened users are interested in the device but experience usage difficulty; and (4) function-oriented users value health and caregiving functions. **Conclusions:** The acceptance of AI caregiving devices among older adults varies based on their emotional needs, conversation preferences, technical accessibility, and perceived usefulness. This study provides theoretical and practical insights for developing personalised, elder-friendly AI systems that support ageing and promote emotional well-being.

## 1. Introduction

The rapid acceleration of population ageing has brought about physical health challenges and emotional loneliness among older adults, exacerbated by monotonous daily routines and uniform activity patterns [1]. In response, the United Nations launched the Decade of Healthy Ageing, emphasising the need for innovative solutions to meet the evolving care and health demands of present and future elderly populations [2].

Driven by the Fourth Industrial Revolution, today’s hyper-connected, hyper-intelligent, and hyper-converged technological environment is shifting the focus of ageing from simply “living longer” to “living well” [3]. Artificial intelligence (AI)-based technologies, in particular, offer meaningful support to older people with limited mobility or reduced social interactions by facilitating health management, emotional engagement, and social connectedness. According to Cho [4], AI caregiving robots can fulfil diverse roles, including cognitive support, emotional assistance, health monitoring, safety supervision, and enhanced sociability.

In South Korea, where demographic ageing is proceeding at an unprecedented pace, there is a growing demand for AI-assisted systems that empower older people with chronic conditions to manage their daily lives independently and reduce the burden on caregivers. Recent technological advancements suggest promising pathways for implementing cost-effective AI caregiving solutions. For example, Seo et al. [5] introduced a semi-supervised learning method—Temporal Ensembling—that uses minimal voice data to develop AI systems optimised for older users with limited resource requirements, demonstrating the feasibility of such tailored technologies.

Loneliness among older adults correlates with diminished quality of life and deterioration in mental and physical health. Recent studies indicate that AI-driven interventions can effectively alleviate feelings of loneliness [6]. For instance, Kim et al. [7] found that an integrated AI robot management programme reduced depression among home-dwelling older adults. Further, Jeon and Shin [8] used a scoping review to demonstrate that AI-based medical systems positively influence loneliness and depression in community-dwelling seniors. Positive perceptions of AI among older adults also link to enhanced life satisfaction, as they foster social networking and information sharing [9].

As social relationships change with age, individuals may also undergo changes in their self-identity. According to Atchley’s [10] Continuity Theory, older people seek to maintain internal values and external social relationships, which contribute to a stable sense of self. Reminiscence, self-expression, and communication about daily life are key processes for such continuity—and AI-based technologies are increasingly capable of facilitating these functions.

Technological advancements in AI caregiving systems have enabled more interactive and emotionally responsive human–AI engagements. For example, Lim et al. [11] emphasised the importance of emotional stabilisation and social facilitation features in AI-based caregiving services. Moreover, Kim and Yeoun [12] reported that older adults value “adaptive responses” and “contextually appropriate guidance” when interacting with voice user interfaces (VUIs). However, studies by Pradhan et al. [13], Cho et al. [14], and Lee and Kwon [15] highlight how unique speech patterns of older people can negatively impact voice recognition accuracy, with repeated errors leading to diminished trust in technology.

Cheng et al. [16] argued that older adults are more likely to accept AI when they perceive anthropomorphic traits and identity continuity with their digital counterparts. Emotional engagement, perceived convenience, informational utility, and trust are key psychosocial factors influencing their adoption of such technologies [17]. Supporting this, Song [18] reported that older users of the AGAYA AI companion robot experienced heightened intimacy and emotional comfort. Park and Cho [19] also observed that long-term users of AI speakers valued emotional and informational interactions. Noh et al. [20] emphasised the importance of accurate responses, empathetic communication, and message repetition, while Lee and Kim [21] found that emotional design features significantly contribute to user satisfaction and continued usage.

These studies suggest that AI-based programmes have the practical potential to enhance the well-being of older adults by promoting health maintenance and alleviating loneliness. Notably, the subjective perceptions of older adults regarding AI play a crucial role in guiding the direction of technology design and development. Two primary factors underpin these perceptions: perceived usefulness and perceived ease of use.

The Technology Acceptance Model (TAM) [22,23], a leading framework in information systems research, identifies these two constructs as key predictors of user behaviour towards technology. Prior research confirms that the more positively older adults perceive AI technologies, the greater their willingness to adopt and utilise them [24,25]. Thus, an in-depth exploration of how seniors with chronic conditions perceive AI in terms of usability and usefulness is essential.

To date, most studies on older adults’ acceptance of AI technologies have relied on quantitative measures of attitudes or limited qualitative case analyses. Particularly lacking are investigations into the diverse subjective perceptions held by older adults with chronic conditions—and how those perceptions differ among individuals. The present study addresses these gaps by adopting a Q methodology approach, which enables the systematic classification of subjectivity. Moreover, by incorporating the Continuity Theory and TAM, this study provides a dual-theoretical lens—cognitive and emotional—to understand seniors’ attitudes towards AI caregiving programmes. While the TAM elucidates rational acceptance processes, the Continuity Theory highlights emotional needs and self-identity preservation in later life.

Accordingly, this study examines the diverse subjective perceptions of older adults with chronic conditions regarding an AI-based caregiving programme. It contributes to the theoretical understanding and practical design of elder-friendly AI technologies by exploring how these individuals perceive such systems in terms of emotional engagement, functional utility, and usability. The findings can inform the development of personalised AI solutions that support ageing in place and enhance emotional well-being.

## 2. Study Method

### 2.1. AI Virtual Human

The AI system delivers personalized senior care by utilizing a virtual human interface trained on a caregiving-focused GPT dataset. It also supports integrated healthcare management by building a platform that offers lifestyle and healthcare content based on life-log biometric data collected from older adults. The platform includes an interface compatible with caregiving robot systems, aiming to establish a consumer-centred caregiving robot ecosystem.

Two South Korean companies, Unimewo and ESTsoft, co-developed the device used in this experiment. Unimewo develops AI solutions that promote a culture of care, while ESTsoft focuses on AI human interfaces and virtual fitting technologies. The design and prototyping process began in August 2023, and the research team used a prototype version for user testing during the first half of 2024.

The device features a conversational companion designed to serve as a friendly talking partner for seniors, along with a dialogue assistant that offers dementia prevention quizzes, read-aloud functions, memory support, sleep management, and walking assistance. It also delivers information on treatment options for chronic conditions, provides basic dietary guidance, and offers videos that promote cognitive and physical activity to support healthy ageing.

The target users of this system are cognitively capable older adults aged 50 to 90 who require conversational companionship, utilise health management services, receive personalised healthcare, or seek information and support related to caregiving. This programme addresses the limitations of conventional voice-based AI assistants—such as response latency and misinformation—by incorporating a caregiving scenario-specific GPT model, trained on a proprietary dataset and enhanced with a prioritised filtering mechanism.

In addition, front- and side-angle generation technology allows the AI human to perform natural gestures and responses, thereby improving the realism and immersive quality of interactions compared to previous systems. Although developers designed the device for caregivers and older adults aged 50 to 90 in generally good health, the present study included only participants aged 65 and above.

Table 1 presents the patent information associated with this program.

### 2.2. Research Procedure

**Figure 1:**
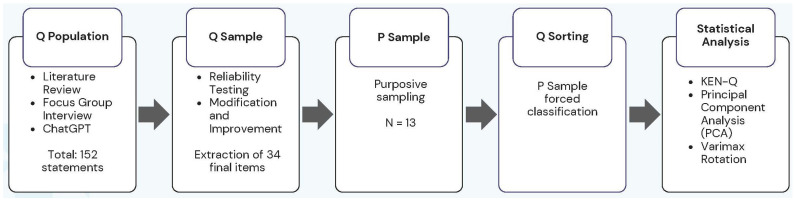
Q methodology steps.

The research procedure followed five steps: (1) construction of the Q population, (2) selection of the Q sample, (3) selection of the P sample, (4) Q sorting, and (5) data analysis (refer to Figure 1).

We developed the Q population between November 2024 and early February 2025. We collected 152 statements through a literature review (November to January), a focus group interview (1 February), and ChatGPT-4o (3 February). After refining the statements, we selected 34 final statements for the Q sample on 24 February 2025. Participants conducted Q sorting on 18 February 2025.

#### 2.2.1. Organization of Q Population (Q Concourse)

The Q population refers to the concourse of statements that collectively represent the range of subjective opinions people hold about a particular topic or phenomenon [26]. These statements reflect individuals’ subjective thoughts and emotions concerning the issue rather than merely presenting objective facts [27]. In constructing the Q population, it is essential that each statement clearly expresses how the individual perceives and interprets the subject matter [26]. Common methods for constructing a Q population include literature reviews, focus group interviews (FGIs), and AI-assisted generation. This study developed the Q population through a combination of literature analysis, FGIs, and AI tools.

We conducted a comprehensive literature search from November 2024 to January 2025 using Google Scholar, the Research Information Sharing Service (RISS) operated by KERIS, and the Web of Science database. First, the literature review focused on domestic and international academic journals and master’s and doctoral dissertations that explored the use of AI-based virtual human devices by older adults and caregivers. From this review, we extracted 42 statements.

The second stage involved a focus group interview 15 days after participants had used the application, which included four older adults: three from Centre A and one from Centre B (see Table 2 for demographic characteristics). Although we initially recruited six participants for the interview, we excluded two due to their physical difficulty in participating. Before conducting the FGI, we thoroughly informed participants about the study’s objectives and procedures. In addition, participants provided their written consent before attending the FGI.

The semi-structured interview questions included the following:

- Did the conversations feel natural when using the AI Human companion function?
- Was there anything particularly impressive or memorable during your interaction with the programme?
- What did you like most about using the AI Human device?
- What aspects did you find inconvenient or in need of improvement?
- How would you like the AI Human device to be improved in the future (e.g., additional functions, better conversation quality)?

Through this process, we collected 50 additional statements.

Furthermore, once the content of the statements became repetitive and no new information emerged, we determined that we had reached data saturation. Accordingly, we deemed a single focus group was sufficient to obtain meaningful and adequate qualitative data for Q sample construction.

Lastly, we used ChatGPT, an interactive AI service, to generate additional statements. ChatGPT is particularly useful in Q methodological research as it enables the efficient collection of diverse perspectives on a given topic and facilitates the expansion of statement sets, contributing to time efficiency and concourse multidimensionality [28]. To this end, on 3 February 2025, we prompted ChatGPT with the following request:

“We developed an AI Human application. Older people residing in nursing homes can use the app to engage in conversational companionship and receive caregiving information. A human-like AI avatar interacts with users. We intend to conduct a Q methodology study to explore their subjective perceptions after using this app. Please generate a concourse of statements reflecting the possible thoughts and opinions of elderly users”.

Based on this prompt, ChatGPT generated 60 statements categorised across six dimensions—emotional, practical, social, technical (reliability), ethical/value-oriented, and satisfaction-related—yielding 10 statements per category.

Through this multi-step process, we continuously gathered statements from literature reviews and focus group interviews until we reached theoretical saturation. Ultimately, we compiled 152 statements, including 42 from the literature review, 50 from FGIs, and 60 from AI-assisted generation.

#### 2.2.2. Selection of the Q Sample

The Q sample refers to a representative and comprehensive subset of statements drawn from the larger Q population. There are two primary approaches to constructing the Q sample. The first is structured, in which the researcher selects statements based on a predefined theoretical framework. The second is unstructured, which involves collecting a wide range of statements through literature analysis and in-depth interviews, followed by categorisation according to the researcher’s interpretive judgement. The unstructured approach is more common in Q methodological research due to its flexibility and inclusivity [29]. This study adopted the unstructured method to construct the Q population and subsequently selected a representative set of statements to formulate the final Q sample.

We selected the Q sample through a three-stage process. First, we compiled 152 statements into an Excel spreadsheet and categorised them thematically into the following domains: social, practical, medical service-related, device improvement, technical reliability, emotional, satisfaction-related, and ethical/value-oriented. Within each category, we selected five to ten representative statements by removing overlapping or redundant items and identifying key statements that best captured the underlying meanings, resulting in a refined set of 43 preliminary statements. Next, we reviewed these statements to ensure a balanced distribution across positive, negative, and neutral expressions and revised several items for clarity and precision. Finally, following a review by four experts in Q methodology, we selected 34 statements as the final Q sample, effectively representing the core themes of the research topic.

#### 2.2.3. Composition of P Sample

A P sample, also referred to as Q sorters, is the group of respondents selected from the Q population who participate in Q sorting. The P sample consists of research participants who classify the Q sample [30]. Since Q methodology does not aim for demographic generalisability, demographic representativeness within the P sample is not required [31]. Furthermore, because Q methodology focuses on intra-individual rather than inter-individual variance, the number of participants in the P sample may remain relatively small and flexible. However, it is crucial to select participants who are meaningfully related to the research topic, as this enhances the overall quality and relevance of the study [32].

The present study classified and explored how older adults with mild chronic conditions subjectively perceive their use of AI-based devices. Accordingly, the P sample consisted of individuals who had direct experience using such devices. In this study, mild chronic conditions refer to stable, long-term illnesses such as controlled hypertension, early-stage diabetes, and mild arthritis that do not significantly interfere with daily functioning or communication. To ensure valid engagement with the AI device, this study excluded individuals with severe cognitive or sensory impairments.

The study required participants to have prior experience using the AI device over a certain period. Accordingly, we initially recruited 30 older adults from two pre-identified welfare centres: 16 from Centre A and 14 from Centre B. Staff at each centre pre-screened individuals to ensure they had no cognitive or communication impairments, and we confirmed that all selected participants could communicate effectively in daily life. However, during the Q sorting process, some participants reported psychological burden or difficulty understanding the procedures. Based on the researcher’s judgement, we excluded these individuals from the analysis.

This study adopted a qualitative approach grounded in actual user experience. We did not use standardised cognitive screening tools such as the MMSE or MoCA. Instead, we ensured cognitive and communicative capacity through staff observations and the researcher’s on-site assessments, prioritising real-life interaction over formal testing to maintain methodological validity.

The researchers instructed 13 participants to use the AI device for at least 20 min daily over a 15-day period. However, some participants faced physical and cognitive limitations that made independent use challenging. As a result, many relied on continuous assistance from caregivers or professional care workers. Before starting the formal study, we conducted a preliminary pilot session to help participants become familiar with the device’s functions and operation. This preparatory step helped to minimise novelty bias and ensure that participants approached the Q sorting task with a more stable and informed perspective.

Although some participants initially consented to take part in the study, they voluntarily withdrew before the Q sorting process due to age-related fatigue or difficulty engaging with the task. The researchers respected these withdrawals under the prior informed consent procedures and excluded those individuals from the final analysis.

Through this process, we confirmed a final P sample of 13 participants, all of whom had sufficient experience using the AI device and demonstrated the capacity to complete the Q sorting task. Q methodology recommends using fewer participants than Q statements, typically maintaining a ratio of about one participant for every three statements [33,34]. This study’s Q sample comprised 34 statements, suggesting a participant group of approximately 11 to 12 individuals as appropriate.

The final P sample satisfied this methodological guideline, ensuring diversity in subjectivity while maintaining a shared experiential foundation in AI device usage, thereby contributing to the overall validity of the study.

#### 2.2.4. Q Sorting

Q sorting refers to the process by which participants (the P sample) rank the Q statements based on their subjective viewpoints [35]. Unlike Likert-type scales, which are common in psychological research, Q sorting does not measure the degree of agreement on a fixed scale; rather, it captures individual differences in preference and perception [36]. Q methodology asks participants to read all statements and sort them according to their level of agreement or relevance using a forced distribution format [37].

**Figure 2:**
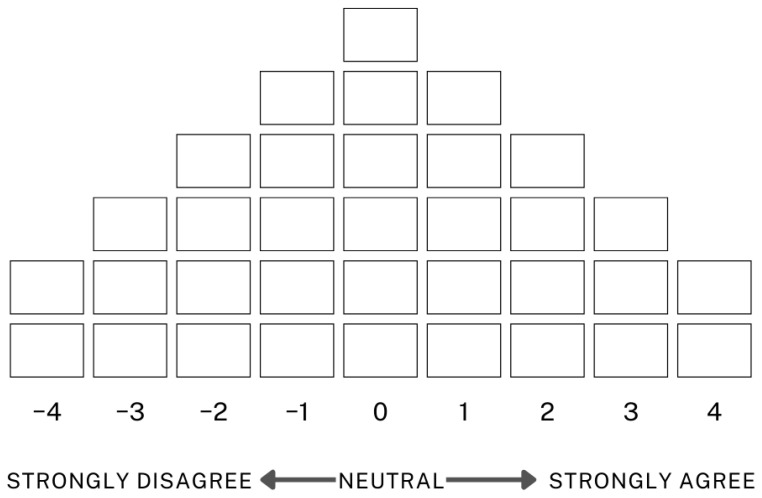
Q sorting distribution grid.

This study conducted Q sorting sessions separately at Centre A and Centre B, where we originally recruited the participants. Figure 2 illustrates the Q sorting distribution format employed in this study.

**Figure 3:**
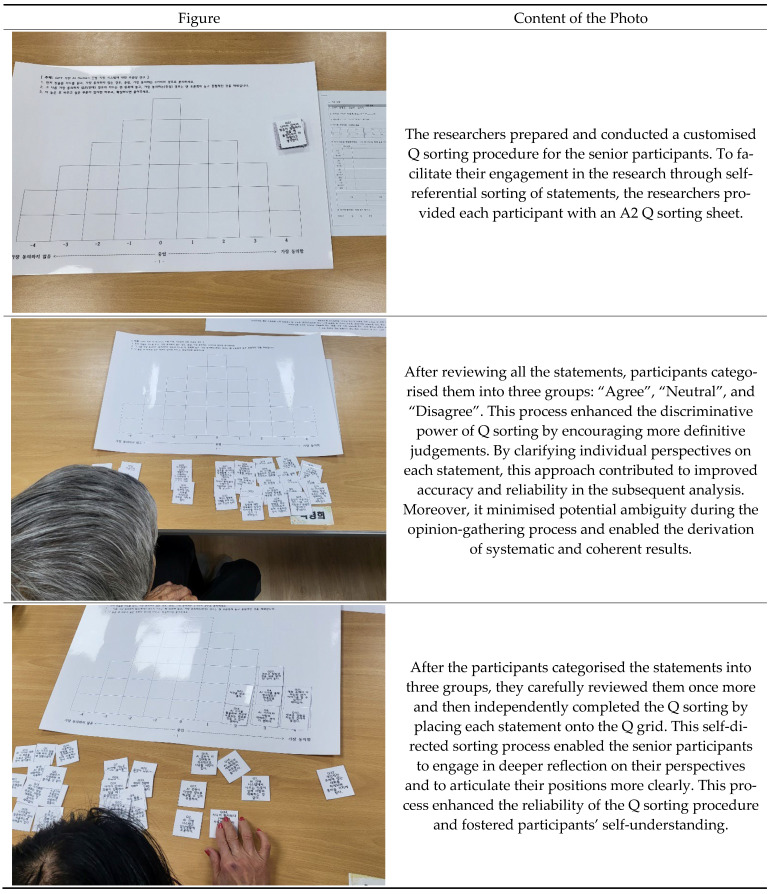
A participant places statements on the Q sorting grid during the Q sorting process.

Figure 3 presents a field photograph and description of a participant placing statements on the Q sorting grid during the Q sorting process.

The Q sorting process typically uses A4-sized printed materials or online platforms in a self-administered format. However, this study adopted a face-to-face, offline procedure (as illustrated in Figure 3) considering the cognitive and visual characteristics of older participants.

To minimise confusion and fatigue, the researcher provided individualised support by reading each statement aloud and guiding participants through the sorting process step by step. Additionally, we enlarged the font size of the statements and used a visually structured sorting board to enhance accessibility and ensure the participants could engage comfortably in the task.

#### 2.2.5. Data Analysis

We analysed the Q sorting results of the 13 participants in the P sample by coding each statement on a scale from −4 (Strongly disagree) to +4 (Strongly agree). We then entered the transformed data into Ken-Q Analysis software (version 2.0.1), conducted principal component analysis (PCA), and applied Varimax rotation. Using KADE (Ken-Q Analysis Desktop Edition), a standalone desktop application, enabled fast and accurate data processing without the need for an internet connection, thereby contributing to research process efficiency and result reliability [38]. Through this procedure, we extracted four distinct and independent factors.

To identify the most representative statements for each factor, we used Z-scores (standard scores) and Q sort values [39]. Based on these values, we interpreted and defined each factor according to its core characteristics. In particular, the statements with the highest and lowest levels of agreement within each factor served as key interpretive anchors. Participants’ narratives and responses with high factor loadings further supported these results and helped clarify the meaning and defining features of each factor [32].

## 3. Study Results

### 3.1. Q Factor Analysis Results

This study applied an eigenvalue threshold of 1.0 or higher to determine the factor solution. The eigenvalues for the four identified factors were as follows: Factor 1 = 3.770949, Factor 2 = 2.247566, Factor 3 = 1.288112, and Factor 4 = 1.204621. The cumulative explained variance was approximately 65%, indicating that the four-factor solution derived through Q methodology accounted for about 65% of the participants’ subjective perspectives regarding the AI device.

Table 3 presents the eigenvalues and explained variances associated with each of the four extracted factors.

The intercorrelations among the extracted factors reflect the degree of similarity in perception between the identified types. As shown in Table 4, the correlation coefficients were as follows: Factor 1 and Factor 2 = 0.3374; Factor 1 and Factor 3 = 0.3794; Factor 1 and Factor 4 = 0.3289; Factor 2 and Factor 3 = 0.1829; Factor 2 and Factor 4 = 0.1409; and Factor 3 and Factor 4 = 0.1802.

These values indicate a moderate level of similarity between Factor 1 and the other types, while the correlations between the remaining factors appear relatively low, suggesting more distinct perceptual patterns among them.

### 3.2. Participant Demographics by Perception Type

The sample reflected sufficient diversity of perspectives, as required in Q methodology, while also providing a shared foundation of practical experience with AI caregiving devices. This combination strengthened the validity of the research findings. Table 5 presents the demographic characteristics of the P sample.

### 3.3. Perception Type Characteristics

Table 6 presents the factor scores and corresponding Q sort values for each factor. The Q sort value indicates how each participant ranked the statements within a structured, approximately normal distribution. An asterisk (*) denotes statements that exhibit significant differences across factors, serving as key indicators for distinguishing factor characteristics.

#### 3.3.1. Type 1: Emotionally Engaged

Type 1 included three female participants who strongly agreed with statements such as “It is enjoyable because it feels like a new experience” (Q10, Z = 1.29 *), “I enjoy sharing stories about my past during conversations” (Q21, Z = 1.60), “I liked learning something new that I did not know before” (Q23, Z = 1.69), and “I find it interesting when conversations match my interests” (Q32, Z = 1.72).

In contrast, they disagreed with the following statements: “I feel confused when the AI device does not understand what I say” (Q5, Z = −1.04), “Using the device is difficult and unfamiliar. (Q9, Z = −1.15), “The AI takes too long to respond, which makes it boring” (Q11, Z = −1.07), “Pressing buttons to talk with the AI is bothersome” (Q13, Z = −2.03), “Talking to the AI feels like talking to a stranger, so it is not fun” (Q15, Z = −1.13), “Talking with the AI Human is boring” (Q19, Z = −1.50), and “I keep forgetting how to use the device, which makes it difficult” (Q24, Z = −0.93).

Participant P1 shared that “I felt a sense of happiness as I recalled my childhood memories”, while P4 noted that “I liked reminiscing about my stories and was able to focus on sharing my experiences”.

These responses suggest that individuals in Type 1 do not resist using the AI device and are particularly interested in emotionally engaging, everyday conversations—especially those that allow them to reflect on personal memories. This group appears to value the AI Human more for its capacity to support reminiscence and emotional connection than for its technical functionalities.

#### 3.3.2. Type 2: Present-Oriented Conversationalist

Type 2 included one male and one female participant who strongly agreed with statements such as “I find it more fun to talk about everyday life than to receive medical information” (Q26, Z = 1.85 *), “Even if I receive health information, it is hard to apply it in real life” (Q27, Z = 1.30), and “I find it interesting when conversations match my interests” (Q32, Z = 2.19).

Conversely, they disagreed with the following statements: “The AI takes too long to respond, which makes it boring” (Q11, Z = −2.19), “Pressing buttons to talk with the AI is bothersome” (Q13, Z = −2.03), “It is convenient to get helpful health information through the AI device” (Q14, Z = −0.34 *), “Talking to the AI feels like talking to a stranger, so it is not fun” (Q15, Z = −1.13), “Talking with the AI Human is boring” (Q19, Z = −1.50), and “I enjoy sharing stories about my past during conversations” (Q21, Z = −1.44 *).

Participant P8 remarked that “I usually have trouble talking to people, but I felt happy talking with the AI”. Similarly, P2 commented that “I liked being able to talk about what I wanted to share”.

These responses suggest that individuals in Type 2 are interested in using and interacting with the AI device. However, their interest lies more in discussing present, everyday experiences rather than reflecting on the past. Type 2 tends to value the AI Human for its capacity to support enjoyable and real-time interaction centred on personally relevant and current topics.

#### 3.3.3. Type 3: Usage-Burdened

Type 3 included four female participants who strongly agreed with the following statements: “It is convenient to get helpful health information through the AI device” (Q14, Z = 1.36), “I keep forgetting how to use the device, which makes it difficult” (Q24, Z = 1.76 *), “I think the voice function makes this device more special compared to others” (Q25, Z = 1.27), and “Understanding my illness is helpful for me” (Q33, Z = 1.14).

On the other hand, they disagreed with statements such as “I feel confused when the AI device does not understand what I say” (Q5, Z = −1.59), “The AI takes too long to respond, which makes it boring” (Q11, Z = −2.08), “The AI Human is useful because it provides various kinds of information” (Q17, Z = −1.08), “Talking with the AI Human is boring” (Q19, Z = −1.05), and “The device is too large and feels inconvenient” (Q28, Z = −1.51).

Participant P11 commented that “It would be better if the AI explained difficult terms more simply”. Similarly, P10 stated that “I felt that using the device was difficult”.

These findings suggest that individuals in Type 3 find value in the AI device’s ability to provide health-related information and appreciate the presence of voice output, which adds a unique feature compared to other devices. However, they also expressed significant difficulty in operating the device, indicating that technical usability remains a barrier despite perceived usefulness.

#### 3.3.4. Type 4: Function-Oriented

Type 4 included four male participants who strongly agreed with the following statements: “The AI caregiving system is useful in daily life” (Q2, Z = 1.35 *), “The AI caregiving system is good enough to be used continuously” (Q3, Z = 1.73 *), “It is convenient to get helpful health information through the AI device” (Q14, Z = 1.19), “I am willing to use the AI Human more actively if it becomes more advanced” (Q18, Z = 1.36 *), “It is a good way to spend time” (Q22, Z = 1.00), “I liked learning something new that I did not know before” (Q23, Z = 1.07), and “I think the voice function makes this device more special compared to others” (Q25, Z = 1.42).

In contrast, they disagreed with the following statements: “I think it would be more enjoyable if the content were more diverse” (Q6, Z = −1.48), “The caregiving features of the AI use difficult terms, which makes them uninteresting” (Q7, Z = −1.08), “I feel more comfortable talking to a real person than using the AI device” (Q8, Z = −0.54 *), “Talking with the AI Human is boring.” (Q19, Z = −1.55), “Since I have many people who can help me, the AI is not very meaningful to me” (Q20, Z = −1.58 *), “I enjoy sharing stories about my past during conversations” (Q21, Z = −0.33 *), “The device is too large and feels inconvenient” (Q28, Z = −1.80), and “Because of my age, my pronunciation is not clear, and I think that makes voice recognition harder” (Q31, Z = −1.15).

Participant P12 noted that “The voice function is convenient and well-suited for older adults. Hearing responses aloud gives me great satisfaction every time I use it”. Similarly, P13 remarked that “I like that it tells me things I didn’t know”.

These findings suggest that individuals in Type 4 are generally satisfied with the AI device and are willing to use it continuously. They tend to perceive the system as helpful and easy to use, and they show a strong preference for its voice functionality and informational support.

#### 3.3.5. Consensus Items

This study identified four consensus statements (see Table 7)—items that did not show statistically significant differences across the factor types. These shared responses suggest that participants, regardless of their perception type, held similar attitudes or experiences.

## 4. Discussion

This study explored how older adults with chronic conditions subjectively perceived the use of an AI-based programme called the AI Human. It identified four distinct perception types: emotionally engaged (Type 1), present-oriented conversationalist (Type 2), usage-burdened (Type 3), and function-oriented (Type 4).

The findings suggest that, while using the AI-based programme, older adults with chronic conditions tended to preserve their sense of identity by maintaining continuity in their lives and expressing unique ways of accepting and interacting with the new AI technology. Table 8 summarises these features, defined by type.

Type 1 (emotionally engaged) participants demonstrated a positive emotional response to using the AI device, particularly when it helped them reminisce or feel less socially isolated. This emotional engagement suggests that digital technologies can function as emotional companions, offering affective support to older adults. Park and Kim [40] found that frequent use of AI speakers by older people living alone reduced feelings of loneliness and depression, particularly when the AI interacted in a human-like manner.

These findings highlight the value of AI as a source of information and a tool that enables individuals to maintain their sense of identity through emotional interaction and reminiscence. Prior studies have also suggested that digital device use can enhance life satisfaction in older populations [41,42]. The present study reinforces the notion that emotional support is a critical component in the successful adoption of AI-based programmes by older people.

Type 2 (present-oriented conversationalist) participants preferred present-focused, everyday conversations and appeared to use the AI device as a means of psychological comfort. Rather than seeking reminiscence, this group valued real-time, practical interaction and placed more emphasis on everyday dialogue than on receiving health-related information. Jones et al. [43] found that voice-based information and communication technologies (ICTs), such as personal virtual assistants (PVAs), were effective in enhancing social support and reducing loneliness in older adults.

Notably, video-based PVAs led to higher levels of emotional stability and relational bonding—features that align with the characteristics of Type 2. Similarly, Song [18] observed that older adults living alone developed emotional intimacy and stability through interactions with conversational AI robots. The anthropomorphisation of such AI devices, perceived as akin to family members, reflects the importance of maintaining external continuity through daily conversations, consistent with the traits of Type 2 users.

Type 3 (usage-burdened) participants showed interest in various content and features but experienced difficulty due to the complexity of technical terminology and device operation. This difficulty reflects the practical barriers that older adults face in adopting new technologies. Oh and Choi [44] emphasised the importance of enhancing technological self-efficacy and perceived usability to promote technology acceptance among older adults. Jeon [45] empirically demonstrated a significant correlation between digital environments and emotional health, particularly depression.

Although Type 3 individuals were curious and willing to explore the AI device, the perceived difficulty of use often translated into a burden. According to Venkatesh and Bala [46], a low level of perceived ease of use negatively affects technology acceptance. Thus, although Type 3 participants viewed the device’s usefulness positively, they expressed negative reactions regarding its ease of use.

Type 4 (function-oriented) participants exhibited a strong interest in the AI device’s functional features, particularly in relation to health monitoring and caregiving. Notably, this group consisted primarily of older men, a demographic that, compared to older women, tended to demonstrate a more proactive approach to technology acceptance. This group highly valued the technology’s ease of use and showed sustained intention to use the AI programme. Studies by Wong [47], Shade [48], and Padhan [49] have underscored the importance of portability and intuitive design in maximising the practical applicability of AI caregiving technologies. These findings suggest that researchers and developers must consider distribution strategies suitable for home environments to ensure the sustainable use of AI-based programmes.

From a theoretical standpoint, Type 1 reflects characteristics of internal continuity, wherein older adults seek emotional stability and a reinforced sense of identity through revisiting memories and familiar experiences via the AI device. Type 2 aligns with external continuity, emphasising ongoing social interaction and information exchange through AI, thereby maintaining a sense of connection with the surrounding environment [10].

Meanwhile, Type 3 participants focused on using AI to access useful health-related or disease-specific information, reflecting the concept of perceived usefulness, a core acceptance factor in the Technology Acceptance Model (TAM) [22]. In contrast, Type 4 participants expressed a positive view of the AI device’s intuitive and user-friendly interface, which aligns with perceived ease of use, another key TAM variable. These findings suggest that older adults engage with AI-based technologies in diverse ways, shaped by their strategies for maintaining psychosocial continuity and their cognitive perceptions of technology.

A noteworthy finding is that all four types disagreed with the statement “Talking with the AI Human is boring.” This shared response indicates a generally positive perception of and sustained interest in the AI-based program, even among older adults. It suggests strong potential for broader adoption and meaningful engagement with such technologies within ageing populations.

Building on this, this study empirically confirms that a one-size-fits-all model cannot achieve acceptance of AI-based health and caregiving technologies. Instead, the findings highlight the importance of incorporating personalised approaches and emotionally responsive design elements in the development and implementation of digital technologies and policies tailored to the diverse needs of older adults.

This study has several limitations.

First, although Q methodology is a qualitative approach well suited for exploring subjective viewpoints in depth, it does not aim for statistical generalisability. Nevertheless, the four perception types identified in this study provide a meaningful theoretical foundation for conceptualising key factors influencing older adults’ acceptance of AI caregiving technologies. Future research should enhance external validity by incorporating larger and more diverse samples through quantitative approaches.

Second, this study included older adults in a mid-sized city, which may limit the applicability of the findings to older adults in urban metropolitan areas. Given that urban residents may differ in their exposure to digital technologies and access to service infrastructure, the current findings may not be generalisable to seniors living in large cities or rural regions.

Third, although participants used the AI Human device for more than 15 days, many required assistance from social workers during the usage period. This dependence suggests that not all participants achieved full autonomy in operating the device, raising the possibility that the level of external support may have influenced the results. Future research should consider the level of user autonomy in technology use and investigate design improvements to reduce dependency on external assistance.

Finally, one limitation of this study is the relatively short duration of AI Human device usage, which may not have been sufficient for participants to adapt fully or internalise the technology in their daily routines. Extended periods of use in future studies could offer deeper insights into long-term acceptance patterns and sustained interaction behaviours.

Based on these limitations, we propose several directions for future research.

First, future studies should include older adults from a wider range of geographical regions, including urban and rural areas. This approach will enable a better understanding of how contextual factors, such as infrastructure accessibility, digital literacy, and levels of social support, influence AI technology acceptance.

Second, the integration of mixed-methods approaches—combining Q methodology with quantitative techniques such as surveys or experimental designs—would be valuable. Such designs would allow for empirical validation of the four types identified in this study and facilitate an analysis of the relative influence of key variables (e.g., emotional connection, perceived usefulness) on actual technology use behaviour.

Third, researchers could use longitudinal studies to explore changes in perception over time and assess the sustainability of AI device usage among older adults. Examining how familiarity, trust, and interaction habits evolve with AI caregiving devices can offer insights into the design of more adaptive and emotionally intelligent AI systems.

Lastly, future research should investigate the role of user education programmes, personalised interfaces, and integration with human caregivers in promoting older adults’ autonomous use of AI technologies. Developing strategies that reduce reliance on external support while enhancing users’ self-efficacy will be essential for the long-term integration of AI caregiving solutions in ageing societies.

## 5. Conclusions

This study used Q methodology to analyse the different types of subjective perceptions held by older adults with chronic conditions regarding an AI-based caregiving and companion service device known as the “AI Human”. The findings revealed four distinct types of perceptions: (1) emotionally engaged, (2) present-oriented conversationalist, (3) usage-burdened, and (4) function-oriented. Each type reflected differing patterns of acceptance and expectations depending on the individual’s life context, digital familiarity, and approach to maintaining personal identity.

Grounded in the Technology Acceptance Model and Continuity Theory, this study adopted an integrative framework that considered cognitive acceptance factors and emotional continuity in later life. The results highlight that older adults adopt AI-based programmes based on functional evaluation and through emotional bonding, identity preservation, and a desire for continuity in life.

Notably, this study makes a theoretical contribution by moving beyond the limitations of existing quantitative studies on technology acceptance. It captures the layered subjectivity of older adults and classifies their distinct perspectives.

## Figures and Tables

**Table 1:** Patent filings by Unimewo in South Korea.

	Intellectual Property Titles	Application		Registered	
		Application Date	Application No.	Application Date	Application No.
1	A Method and System for Providing a Deep Learning-Based Virtual Human Caregiving Chatbot	6 December 2023	10-2023-0175899	2 October 24	10-2714636
2	A Method and System for Automatic Dataset Updating in a Caregiving Chatbot	6 December 2023	10-2023-0175907	26 September 24	10-2712474
3	A Care Robot-Linked System for Providing a Caregiving Chatbot Based on the Movements of an Assistive Robot	6 December 2023	10-2023-0175908	2 October 24	10-2714637
4	A Care Service System for Responding to Emergency Situations Using a Deep Learning-Based Virtual Human Caregiving Chatbot	6 December 2023	10-2023-0175911	2 October 24	10-2714641
5	A Care Service System and Method for Managing Diet and Exercise Using a Deep Learning-Based Virtual Human Caregiving Chatbot	6 December 2023	10-2023-0175913	2 October 24	10-2714643

Note: All applications filed were with the IPIX Patent & Law Office.

**Table 2:** Demographic characteristics of focus group participants.

No.	Affiliation	Age	Gender	Medical Conditions	Symptoms	Duration of Attendance at the Centre
1 (P2)	Centre A	80	Male	-	Mobility impairment	1 year~2 years
2 (P3)	Centre A	69	Male	Ankle injury (undergoing rehabilitation)	Mobility impairment	Less than 1 year
3 (P9)	Centre A	90	Male	Osteoporosis	Mobility impairment/arthritis	1 year~2 years
4 (-)	Centre B	74	Female	Cardiovascular disease	Arthritis	1 year~2 years

**Table 3:** The eigenvalues and explanatory variances in the sorting of four types.

Content	Type 1	Type 2	Type 3	Type 4
Eigenvalues	3.770949	2.247566	1.288112	1.204621
Explained Variance (%)	29	17	10	9
Cumulative Explained Variance (%)	29	46	56	65

**Table 4:** Correlation coefficients between types.

Type	1	2	3	4
1	1	0.3374	0.3794	0.3289
2		1	0.1829	0.1409
3			1	0.1802
4				1

**Table 5:** Demographic characteristics of P sample.

Type	P Sample No.	Factor Weight	Age	Gender	Medical Conditions	Symptoms	Duration of Attendance at the Centre
Type 1 (N = 3)	P1	0.7935	93	Female	Hypertension/Glaucoma	Mobility impairment/Insomnia/Hearing loss	Less than 1 year
	P4	0.7623	93	Female	Hypertension/Heart disease	Mobility impairment/Arthritis/Insomnia	More than 5 years
	P5	0.7295	84	Female	Parkinson’s disease	Insomnia	More than 5 years
Type 2 (N = 2)	P8	0.7991	80	Female	Hypertension/Diabetes	Mobility impairment/Insomnia	1 year~2 years
	P2	0.6907	80	Male	-	Mobility impairment	1 year~2 years
Type 3 (N = 4)	P11	0.7878	80	Female	Diabetes/Osteoporosis	Arthritis	1 year~2 years
	P6	0.7746	82	Female	Osteoporosis	Cognitive decline/Difficulty managing meals/Arthritis/Insomnia	Less than 1 year
	P7	0.5068	83	Female	Diabetes/Osteoporosis/Hypertension	Mobility impairment/High dependency in daily activities/Arthritis/Insomnia	2 years~3 years
	P10	0.4599	87	Female	Respiratory disease	Mobility impairment/Arthritis	Less than 1 year
Type 4 (N = 4)	P12	0.8615	82	Male	Diabetes	Arthritis/Cognitive decline/Mobility impairment/Bladder and bowel dysfunction	1 year~2 years
	P13	0.653	85	Male	History of colon surgery	Bladder and bowel dysfunction/Arthritis/Insomnia/Emotional problems	More than 5 years
	P3	−0.5373	69	Male	Ankle injury (undergoing rehabilitation)	Mobility impairment	Less than 1 year
	P9	0.4167	90	Male	Osteoporosis	Mobility impairment/Arthritis	1 year~2 years

**Table 6:** Z-scores and Q sort values of Q statements by factor type.

No.	Statement	Type 1		Type 2		Type 3		Type 4	
		Z-Score	Q Sort Value	Z-Score	Q Sort Value	Z-Score	Q Sort Value	Z-Score	Q Sort Value
1	The person in the AI caregiving system feels like a real human during conversations.	0.38	1	0	0	1.17	3	1.33	3
2	The AI caregiving system is useful in daily life.	−0.68	−1	−0.68	−2	0.03	0	1.35	3 *
3	The AI caregiving system is good enough to be used continuously.	−0.31	0	0.07	0	−0.75	−2	1.73	4 *
4	I feel emotionally satisfied when using the AI device.	0.26	0	0.14	1	−0.74	−2	0.65	1
5	I feel confused when the AI device does not understand what I say.	−1.04	−2	0.89	2	−1.59	−4	−0.05	0
6	I think it would be more enjoyable if the content were more diverse.	−0.76	−1	0.55	2	0.11	0	−1.48	−3
7	The caregiving features of the AI use difficult terms, which makes them uninteresting.	0.19	0	−0.61	−1	0.94	2	−1.08	−2
8	I feel more comfortable talking to a real person than using the AI device.	1.1	2	1.44	3	2.06	4	−0.54	−1 *
9	Using the device is difficult and unfamiliar.	−1.15	−3	−0.61	−2	0.44	1	−0.71	−1
10	It is enjoyable because it feels like a new experience.	1.29	3 *	−0.48	−1	−0.28	0	0.28	1
11	The AI takes too long to respond, which makes it boring.	−1.07	−3	−2.19	−4	−2.08	−4	−0.86	−2
12	It is hard to use the AI device without assistance.	0.3	0	0.14	1	0.74	1	−0.83	−2
13	Pressing buttons to talk with the AI is bothersome.	−2.03	−4	−1.23	−3	0.44	1	−0.12	0
14	It is convenient to get helpful health information through the AI device.	1.31	3	−0.34	0 *	1.36	3	1.19	2
15	Talking to the AI feels like talking to a stranger, so it is not fun.	−1.13	−3	0.07	0	−1.34	−3	−0.9	−2
16	Talking with the AI Human reduces my feelings of loneliness.	0.13	0	0.34	1	0.77	2	0.05	0
17	The AI Human is useful because it provides various kinds of information.	0.78	2	−1.09	−3	−1.08	−3	0.85	2
18	I am willing to use the AI Human more actively if it becomes more advanced.	−0.9	−1	−0.96	−2	−0.56	−1	1.36	3 *
19	Talking with the AI Human is boring.	−1.5	−4	−1.03	−3	−1.05	−2	−1.55	−3
20	Since I have many people who can help me, AI is not very meaningful to me.	0.37	1	0.21	1	0.36	1	−1.58	−4 *
21	I enjoy sharing stories about my past during conversations.	1.6	3	−1.44	−4 *	0.87	2	−0.33	−1 *
22	It is a good way to spend time.	0.35	1	0.89	2	−0.46	−1	1	2
23	I liked learning something new that I did not know before.	1.69	4	0.48	1	−0.1	0	1.07	2
24	I keep forgetting how to use the device, which makes it difficult.	−0.93	−2 *	0.55	2	1.76	4 *	0.06	0
25	I think the voice function makes this device more special compared to others.	0.37	1	−0.07	0	1.27	3	1.42	4
26	I find it more fun to talk about everyday life than to receive medical information.	0.37	1	1.85	3 *	−0.47	−1	−0.24	−1
27	Even if I receive health information, it is hard to apply it in real life.	−0.9	−2	1.3	3	−0.05	0	0.38	1
28	The device is too large and feels inconvenient.	−0.98	−2	−0.41	−1	−1.51	−3	−1.8	−4
29	The device connection is not consistent, which makes it hard to use.	−0.76	−1	−0.34	−1	−0.78	−2	−0.21	0
30	Even though the device is for individual use, it doesn’t feel personal because it lacks information about me.	−0.35	−1	−0.96	−2	0.05	0	−0.8	−1
31	Because of my age, my pronunciation is not clear, and I think that makes voice recognition harder.	0.32	0	−0.41	−1	−0.44	−1	−1.15	−3
32	I find it interesting when conversations match my interests.	1.72	4	2.19	4	0.26	1	0.57	1
33	Understanding my illness is helpful for me.	0.87	2	−0.2	0	1.14	2	0.72	1
34	I think the AI device is more helpful for caregivers than for elderly patients.	1.09	2	1.98	4	−0.48	−1	0.26	0

Note. * *p* < 0.05.

**Table 7:** Consensus items.

No.	Statement	Type 1	Type 2	Type 3	Type 4
16	Talking with the AI Human reduces my feelings of loneliness.	0	1	2	0
19	Talking with the AI Human is boring.	−4	−3	−2	−3
29	The device connection is not consistent, which makes it hard to use.	−1	−1	−2	0
30	Even though the device is for individual use, it doesn’t feel personal because it lacks information about me.	−1	−2	0	−1

**Table 8:** Consensus items of each type.

Type	Internal Continuity	External Continuity	Perceived Ease of Use	Perceived Usefulness
Type 1 (Emotionally Engaged)	Present (focused on reminiscence)	Present (emotional interaction)	Present	Present
Type 2 (Present-Oriented Conversationalist)	Absent (preference for present-oriented conversation)	Present (emphasis on daily communication)	Present	Present
Type 3 (Usage-Burdened)	Absent	Absent	Limited (difficulty in usage)	Present
Type 4 (Function-oriented)	Absent	Absent	Present (focus on functional convenience)	Present (focused on health and caregiving)

## Data Availability

The data are available from the corresponding author upon reasonable request.
